# Hospital and Institutional News

**Published:** 1914-10-10

**Authors:** 


### HOSPITAL AND INSTITUTIONAL NEWS.

### "THE HOSPITAL'S" SPECIAL NUMBER.

Readers of our Special Number this week will notice that advantage has been taken to place on record a full account of one of the typical base Territorial Hospitals now actively at work. The detailed information collected and the illustrations which accompany it should be invaluable not merely because of the interest centred in these institutions at the moment, but as a document to refer to when the Territorial Hospital which sprang into existence so quickly grows, like the war, a thing of the past. In connection with this article, moreover, it is interesting to read the two illustrated accounts of ambulance trains and ambulance motor-cycles, with the similar problem that they present of ample comfort on the one hand, with, unfortunately, but limited accommodation on the other. The opening of a new volume has also given us the opportunity to direct attention to the tendency of the present day in hospital work, which finds such a remarkable expression in the recent growth of hospital literature, and the causes associated with it. Much interest, we believe, will also centre in the illustrations of home-made furniture which we publish with descriptive accounts; for it is on these minor matters of equipment, small in themselves, on which much money can be well spent, or wasted; and different institutions tend to keep their own "tips" in this department to themselves, and information concerning them is not very easy to arrive at. The subject of hospital economics, with its ever-present problems, provides the subject-matter of another article, and with the War News from the most active of the hospitals we may fairly claim that the range of interest and information in this Special Number is one which will be appreciated by our readers.

### TECHNICAL EQUIPMENT MEANS JOY FOR THE WORKERS.

We desire to have one word with every member of a hospital or institution committee into whose hands the present Special Number of The Hospital may fall. As we have inspected these institutions during the last forty-five years in all parts of the world, we have learnt that efficiency in administration is in exact proportion to the technical knowledge and resulting personal interest which each committee-man takes in the working of his institution. In an important town like Birmingham, or Newcastle, or Liverpool, to take three examples, it is regarded by the leading men of these great cities as an honour to be invited to join the committee of one of the great hospitals. The same keenness is often manifested in the working of even the smallest institutions in country and semi-rural districts, which consequently become splendidly efficient in their own class. It is the object of The Hospital to make the technical and knowledgeable equipment of every committee man throughout the country easy of attainment. Our subscribers include an ever-increasing number of this type of worker.

### WHY "THE HOSPITAL" READERS ARE DILIGENT READERS.

The columns of The Hospital are always open to every worker in the hospital and philanthropic world, when they desire information, or wish to make any point of interest in their own particular field. In this way The Hospital has grown to be regarded as a welcome friend in the households of hospital workers everywhere, and the heartiness of this welcome is extending with the ever-growing numbers of those who serve on hospital committees, as well as to the officers, in exact proportion to their desire to become of practical use in the days of their voluntary service for the sick. We desire at the opening of the twenty-ninth year of our life as the first and oldest hospital journal to grasp the hand of every hospital worker all the world over. Our friends are so world-wide that the post at Christmas brought us greetings from Europe, Asia, Africa, and America, the universality of which was completed in the New Year by numerous letters from residents in Oceania. Words cannot express the warmth of the acknowledgments we desire to offer to hospital workers everywhere. Let us assure them that their contributions and co-operation are of vital importance to The Hospital, for they constitute it the modern newspaper of administrative medicine and institutional life.

### MATTERS OF SPECIAL INTEREST.

We have received most interesting particulars of hospital work with the British Army in the field. These articles are the work of fluent writers who have a thorough mastery of field hospital work, ambulance organisation, and previous experience in war. We have also in hand a plan whereby many more illustrations of the practical side of the work in our hospitals may be given, with the co-operation of those hospital officers who are masters of photography. The work of some of these gentlemen is known for its excellence, and we should welcome offers of co-operation, on a remunerative basis, to ensure our plan being made as representative as possible. We would commend to all workers the contribution on pages 49 and 50, on the subject of "Some Home-made Hospital Furniture," from a very able hospital craftsman holding the office of clerk of the works. We could wish that every craftsman in every great hospital would decide to commence with us the twenty-ninth year of our existence by becoming one of our contributors. The life of a weekly journal is the interest and co-operation of its readers with the Editor. In this way many minds collectively work together with mutual results for good to themselves and the world in which they labour.

### HOSPITAL GARDENS.

We have had the pleasure from time to time to record the beauties and to testify to the material advantages of large and well-equipped hospital gardens. The hygienic value of the hospital garden cannot be too greatly extolled. Gardening to those who love Nature—and what hospital worker is there who does not?—is an education and refreshment to the tired worker. This subject may even prove illuminating when treated with sufficient ignorance and due parsimony, as a letter from a member of the staff of the Cumberland Infirmary in another column demonstrates. Cheerfulness in the sick is the high-road to recovery. The substitution of a wilderness for a garden round hospital wards contributes to a lowered vitality. The hospital garden promotes the return to vigorous life.

### THE EQUIPMENT OF TERRITORIAL HOSPITALS.

The evolution of a Territorial hospital in accordance with Lord Haldane's original scheme has evoked considerable and ever-extending interest amongst the more active workers throughout most of the counties in Great Britain, and especially at Leicester. We had hoped to give an account which exhibits the extent of this interest and its fruitfulness in results for good directly to the Government, to the soldiers for whom the Territorial hospital is established, and no less certainly to the citizens, who devote much time and money in co-operation which cannot fail to produce life-long friendships, and to excite interests in new works for the good of others which will endure. We have received from that indefatigable worker, Mr. H. Johnson, the house governor and secretary of the Leicester Royal Infirmary, a very able contribution on the subject, which affords many interesting proofs of the force of the view here expressed. The large amount of space occupied by the plans and particulars of the 5th Northern General Hospital in this week's issue renders it necessary to hold over until next week an appreciation of the invaluable services rendered by the citizens at Leicester. We shall hope to receive from many hospital workers who have interested themselves in the establishment and equipment of other Territorial hospitals an account of the men and women who have cheerfully devoted themselves to this useful work for the sick and wounded in the war.

### THE VOLUNTARY HOSPITALS AND THE WAR.

The voluntary hospitals are giving invaluable support to the Government by helping them at the outset materially through the provision of a number of beds for the wounded. In the majority of cases wards have been cleared, and in this way accommodation has been provided for a fixed number of our soldiers. In other cases, as at Newcastle-on-Tyne, where the pressure upon the beds was too great to enable the authorities, with due regard to their responsibilities to the civil population, to provide accommodation in existing buildings, the call of the Government for aid has been met in a special manner. With great public spirit the authorities at Newcastle Royal Victoria Infirmary have erected temporary buildings at the back of some of the ward blocks, without interfering with their lighting or hygienic condition. In this way, owing to a previous experience of temporary buildings, erected by Messrs. Corvieson and Son, of Glasgow, which proved a success at Newcastle, the managers have enlisted the same firm to provide excellent wards at small cost, an account of which, with a plan, we hope to give next week. This departure at Newcastle has a special interest from the fact that the administration and work of this infirmary are proverbially good business, and cannot fail to prove of interest to voluntary hospital managers all over the country.

### THE WELSH HOSPITAL.

Despite the fact that the Executive Committee of the Welsh Hospital has limited the present appeal to £25,000, money continues to be sent, and another two beds were endowed last Saturday. The first, or advance party of the hospital, will, according to present arrangements, proceed to Netley on October 19, in order to make final preparations for the reception of patients. It is expected that it will be ready to treat the wounded a week later, when Colonel Sheen will leave for Netley in order to take command.

### A WORKING-CLASS COT IN MANCHESTER.

In July last the "Daisy Day" Committee of Manchester held their second carnival in aid of Ancoats Hospital, in the hope that the proceeds would be sufficient when added to the £720 which they handed over to the hospital authorities last year as the proceeds of the first effort on its behalf. Last week they had the joy of handing over to the hospital committee, at a special meeting convened for the purpose, the handsome sum of £1,500 which, together with last year's amount, made the grand total of £2,220. Councillor J. C. Grime, the chairman of the "Daisy Day" Committee, in presenting the cheque, remarked that considering the greater part of the money was collected in the districts which the hospital served and in the unaristocratic part of Manchester, they ought to be justly proud. The Chairman of the hospital, Mr. Wm. Armitage, in thanking the "Daisy Day" Committee for their generosity, endorsed Councillor Grime's remarks, and pointed out that the hospital derived a large portion of its funds from the working classes. He then invited the "Daisy Day" Committee, before they left the hospital, to inspect the cot, which had been named as follows: "The Daisy Day Cot, endowed by the Daisy Day Committee, per Councillor J. C. Grime, 1914."

### MEDICAL OFFICERS AND THE WAR.

The difficulties experienced by Boards of Guardians in dealing honourably with members of their medical staff who have joined the Forces was exemplified in a case which engaged the attention of the Camberwell Board of Guardians at their last meeting. A fortnight previously they had appointed Mr. Leslie R. Reece, M.R.C.S., L.R.C.P., assistant medical officer at the infirmary and Gordon Road institution, but at the next committee meeting he announced that he was proceeding to Belgium with a Red Cross unit to which he was attached. The board had adopted a resolution that all officers joining any of H.M. Forces should receive half-pay during their absence through military service, but at the time it was adopted they hardly anticipated that it would apply to those who had been in the service of the board such a short period as Dr. Reece, who had been engaged since the outbreak of hostilities. The board, however, had no option but to come to the conclusion that they must abide by their resolution, and pay him half salary, although it is only fair to say that Dr. Reece when before the committee did not suggest in any way that he was entitled to payment. Many authorities who have adopted similar resolutions have found themselves in the same predicament as the Camberwell Board of Guardians, and have had to amend their resolution governing payment to officials with the Forces, so as not to allow it to be taken advantage of.

### THE RED CROSS ON ANNUAL REPORTS.

The Cambridge Town Council has received a communication from the Home Office directing attention to the provisions of the Geneva Convention Act, 1911, which prohibits the use of the Red Cross for business or for any other purpose without first obtaining the authority of the Army Council. The communication states that contraventions of the Act were frequently reported, and suggested that when cases of the use of the Red Cross are brought to the notice of the Town Council inquiries should at once be made as to whether the use of the emblem, "Red Cross," has been officially sanctioned. "With regard to this," writes a correspondent, "in what position are the many hospitals throughout the British Empire which use the Red Cross upon their annual reports and other literature, and also on their collecting-boxes?" What are our readers' opinions?

### SURGEONS FOR SERBIA.

We are informed that the managers of the Serbian Relief Fund are greatly in need at the present time of fully-qualified surgeons, who are prepared to give their services in Serbia on condition that their expenses are paid. The managers, however, are not in a position to offer salaries to intending candidates, but a genuine opportunity exists for all really enthusiastic surgeons who wish to offer their services most usefully at the present time. All inquiries should be addressed to Mr. Bertram Christian, Chairman of the Serbian Relief Fund, 22 Berners Street, Oxford Street, W.

### MENTAL HOSPITALS' STAFFS AND RECRUITING

Since up to the end of September sixteen members of the staff of Croydon Mental Hospital, being either Reservists or Territorials, have joined the Colours, the Chairman of the finance committee has submitted a report stating what provision has been allowed to them. The committee recommended that, while leave of absence without pay should be granted to each member of the staff on military service, allowances equal to half-pay, being not less than 15s. and not more than £2 per week, should be paid to the wives of all married men. In consultation with the Medical Superintendent, the Chairman of the finance committee and the clerk were authorised to make allowances not exceeding those for married men of similar standing to single men with dependants. Contributions to the superannuation fund are to be borne by the visiting committee until the men's return. As regards members of the staff who, not being Reservists or Territorials, wish to enlist, the committee recommended that any who had been in their service three months prior to August 5 should be entitled to the same allowances as those outlined above. It is to be hoped that the allowances made to men who, though bachelors, yet have dependants, will be as liberal as to those with wives, for in the class to which attendants belong there are many cases which deserve such treatment, and often men who have relatives, other than wives, mainly dependent upon them for support.

### FROM CHAPLAIN TO CHAIRMAN AT PORTSMOUTH.

An interesting appointment to the chairmanship of the Portsmouth and Gosport Hospital has been made with the election of the Rev. William C. Hawksley, who has been connected with the hospital in various capacities for a quarter of a century. In acknowledging his election, Mr. Hawksley remarked that nearly twenty-four years ago he had been first appointed chaplain to the institution, and that he had been connected with it almost uninterruptedly ever since. In dealing with the financial position, he humorously expressed the opinion that a hospital which had been cradled at the time of the Crimean War was not likely to be stranded on the rocks because the Kaiser had lost his head. Mr. Hawksley succeeds in the chairmanship Mr. H. R. Pink, who has held the post for three years, during which time he has worked hard to secure the King Edward Memorial Fund and to procure a supply of radium for the hospital. The record of the new Chairman is remarkable not only for its length, but because there are not very many chairmen of hospitals who began as chaplains, nor hospital chaplains who end as chairmen.

### BLOCK FLOOR REPLACED BY LINOLEUM.

In spite of the many different types of floor for hospital purposes now available opinion has not yet decided unanimously in favour of any of them. Thus we find that the recent decision of the board of management of Newton Abbot Hospital to refloor the older part of that institution is to express itself by taking up the block floor now in existence. A layer of cement will replace this, on which will be laid linoleum. It is interesting to note this return to-day to an older and once much-respected type of floor, and in this connection we may quote a sentence or two out of Sir Henry Burdett's Report on the Royal Infirmary, Preston, which appeared last week in our columns. In writing of the equipment of this institution, which he praised highly, he remarked: "The managers have deliberately laid down linoleum throughout the passages and many parts of the institution. This linoleum has stood twelve years' wear and tear, and it looked at the time of our visit as if it had only recently been laid. We have always felt ourselves that linoleum has disadvantages and possibly dangers, though the latter have not yet been demonstrably proved. Still, it presents considerable advantages, and if anyone has any doubt on the subject they had better inspect with care the Preston and County of Lancaster Queen Victoria Infirmary." This is valuable as placing theoretical opinions and practical experience side by side, and will, no doubt, encourage the managers of Newton Abbot Hospital in the return to linoleum, which they have adopted in their institution.

### CONDUCTING AN APPEAL WHILE ON ACTIVE SERVICE.

We have heard of many hospital men being called up or volunteering for active service, but so far only one instance has come to our notice of a hospital officer conducting hospital business from camp. The example concerns the King Edward Memorial Hospital, Ealing, the organising secretary of which, Mr. Walter Barnes, has taken advantage of being in the field with the City of London Yeomanry, London Mounted Brigade, to appeal to the friends of the institution to make an extra effort "so that the funds shall not suffer by the absence of some workers," and while he himself is unable to organise the work. We can hardly believe that an appeal thus based can fail to attract wide sympathy and increased enthusiasm, and commend the method to those hospitals who have responsible officials now in camp or with their regiments still in this country.

### POSITION OF THE HOSPITAL SATURDAY FUND.

Last Saturday the quarterly meeting of the Board of Delegates of the Hospital Saturday Fund was held at Red Lion Square, when Mr. Harry Gower, Chairman of the Council, presided. The Secretary, Mr. A. W. Davis, reported that the receipts to 1 p.m. on that day had reached £15,117, being £837 more than at the same period last year. It would interest the board to know, he added, that the previous week they were £1,007 ahead of 1913 at the corresponding period. It will be recalled that Hospital Saturday is being observed to-day, the 10th inst.

### PANEL DOCTOR'S ATTEMPT TO CHARGE AN INSURED PATIENT.

The panel system now appears to be settling down to the mutual comprehension of both panel practitioners and their patients, and complaints are less frequent than was the case at first. That they still occur, however, is shown by the rebuke recently administered by the Bradford Insurance Committee to a panel doctor, as the result of a discussion of a complaint. The Medical Service Sub-Committee reported that an insured person had informed them that a panel doctor had sent in an account for 25s. for professional services. The Sub-Committee point out that the practitioner did not appear before the Committee to tender his explanation, in spite of a notification requesting him to do so. The Sub-Committee, therefore, recommended that he was not entitled to payment on this account, and that the insured person be requested not to pay it. Mr. E. J. Smith, the chairman, remarked that he did not wish to take the least exception to the report of the Sub-Committee, but since this was the second case of the kind in which this practitioner was involved, and that he had attended on neither, the doctor, in his opinion, was guilty of discourtesy, and the matter should not be allowed to rest there. After some discussion, in which the suggestion that the doctor should be struck off the panel was made and discarded, a resolution was passed expressing the Committee's strong disapproval of the doctor's action, notifying him of it, and advising him in his own interest to comply with the Committee's requirements in future. The resolution also determined that a copy of its terms should be sent to the Panel Committee, together with the doctor's name. The case certainly is a grave one, and Mr. E. J. Smith deserves commendation for the way in which he handled the discussion and for the firm attitude which he took up in reference to the excuse alleged by one speaker that it was absence on holiday which had prevented the doctor's attendance. Cases of attempted charges like the above must be dealt with severely, apart from any other considerations which in this instance aggravated the offence.

### A GREAT SCOTCH PRACTITIONER.

The death of Sir Henry Littlejohn, who was medical officer of health for Edinburgh for many years, removes one of the most striking personalities in the Scotch medical profession. Almost ninety years of age, he preserved to the end the characteristics of his generation in a certain stately courtesy of demeanour, and of his personality in the unconventional traits for which he was noted. It was as far back as 1862 that he became medical officer of health for Edinburgh, the sanitary condition of which called forth a report from him that is still remembered. Well known also for his evidence in many celebrated criminal trials—he filled the Chair of Forensic Medicine in Edinburgh University for eight years—numerous stories gathered about him in the box. Among the causes which he advocated and the prejudices which he indulged was one in favour of cremation and another in strong opposition to tobacco. Honours naturally accumulated with the passage of years, and Sir Henry will live long in that list of Scotch practitioners who are endeared to the student of medical history and to the man with humanity enough to appreciate the mannerisms of a vigorous mind and a sound heart, coupled with high ability and a keen sense of honour.

### THE BATH STATUTORY HOSPITAL EXTENSION.

What has been locally described as the first official ceremony remembered to have been held at the Bath Statutory Hospital took place last week with the opening of a new block. Dr. Preston King, the medical mayor, performed the ceremony, and Dr. R. J. H. Scott, the chairman, in the presence of his colleagues on the City Council, reminded the visitors that the hospital was established between thirty and forty years ago when adequate staff accommodation, which the new block provides, especially for nurses and maids, was not much considered. In the new building there was accommodation for six nurses on the upper floor, in separate bedrooms, and for eight maids, while a sitting-room for each class is provided, and also a bathroom on each landing. Dr. Preston King pointed out that he could speak with intimate knowledge of the institution, as nineteen years ago he had spent seven weeks there as a patient. In those days the conditions were not all they might have been; he looked forward to a covered way between the blocks in the future. The loan for the huts which were built forty years ago for small-pox patients was only for twenty years, and, he added amid laughter, it had been hoped that a fire would burn them down as they were well insured. The stone block was the nucleus of a better hospital to come. The architect of the building is Mr. F. W. Gardiner, and a brass tablet in the corridor records that: "This extension was opened by the Mayor of Bath, Councillor Preston King, M.D., September 30th, 1914. Councillor R. J. H. Scott, F.R.C.S., Chairman of the Statutory Hospital Committee."

### THE RUBBER FLOOR AT GUY'S HOSPITAL.

Last Tuesday afternoon Lady French was present at Guy's Hospital, when, on behalf of the Rubber Growers' Association, she formally presented to the hospital the rubber flooring whose gift from the company has been previously announced in The Hospital. The flooring has been laid in Stephen ward, and its success after a practical trial will be awaited with interest. Fortunately more than one institution's experience with the new material will be available, since, as we have already noted, institutions both in England and Scotland have received a present of it. The material was handed over to the Royal Infirmary, Edinburgh, so lately as the middle of September, and therefore not only will several tests of its quality be eventually available, but, what is also convenient, these tests will have been carried out practically at the same time. Mr. Cosmo Bonsor said that the staff much prefer it to parquet and mosaic.

### DUBLIN HOSPITALS AND THE INSURANCE ACT.

The Board of Superintendence of the City of Dublin hospitals has presented its annual report, which has been issued as a Parliamentary Grey-book. In regard to the Insurance Act, the board complains that subscriptions have generally fallen off, and that the subscribers explain the fact by saying that they do not see why they should pay twice over for the sickness benefits of those in their employment. The three general hospitals under the superintendence of the board have received nothing under the Insurance Act from approved societies, and the report says that even in the case of tubercular patients, for whom 21s. a week is paid by the Insurance Committee, the hospital is out of pocket. In these circumstances, with a debt of over £9,000 in the case of the Meath Hospital, the board draws the conclusion that the voluntary hospitals can be kept going only by Parliamentary grants-in-aid voted in connection with the Insurance Act. These statements are interesting, not because of their novelty, but as indicating an apparent difference in the effects of the Act on Irish hospitals. First as regards the fall in subscriptions. If the word "general," used in respect of the decline, may be taken at its face value, then we may assume that Irish people are more logical than ourselves.

### THE SUGGESTED REMEDY OF GRANTS-IN-AID.

For when subscribing to hospitals, since the passing of the Act, they are certainly paying twice over to supplement that Act's deficiencies, since it cannot be denied that the implication was that medical benefit was to cover more than merely minor ailments of the out-patient department. The Act was trumpeted as if its rare and refreshing fruit did include institutional treatment, while, as we all know so well, the credit of the Act has only been saved by the voluntary hospitals' generous action in receiving insured persons without charge for in-patient treatment. The remedy of grants-in-aid, unless more carefully explained, appeals to us less than our own arguments in favour of a *pro rata* payment for insured persons in special pavilions to be built for their reception. But the time is not ripe for considering these problems at present. The most important effects of the Act from our point of view, however, are its revelation of a large amount of hitherto unsuspected sickness in the country and the detection of patients requiring in-patient treatment who would not have been recommended for it before. And it is these hard facts, rather than new or old arguments, which will force, we believe, the *pro rata* payments as the practical solution upon the Chancellor in time.

### THE SOUTH MANCHESTER CHILDREN'S HOSPITAL

The Local Government Board has now sent its reply to the protest against the proposal of the South Manchester Guardians to build a children's hospital, which was sent to them in the form of a resolution by the Manchester Guardians. The latter's objection, it will be remembered, was on the ground that the scheme was unnecessarily expensive, and at any rate ought not to be proceeded with until the amalgamation of the three Manchester Unions had been carried out. The Local Government Board's reply was that the scheme had already been sanctioned, whereupon the Manchester Guardians decided that, their protest recorded, they would not urge their objection further. The Local Government Board's decision virtually bears out the attitude which we adopted when discussing the question in our issue of September 12 (p. 647), that, namely, the authorities immediately concerned, provided their scheme is open to central approval, ought to be the best judges of the necessity of the new hospital.

### THE BRADFORD NEW INFIRMARY.

A Local Government Board inquiry is being held in the Town Hall, Bradford, on Friday, October 9, into the application by the City Council of Bradford for sanction to borrow the sum of £100,000 for the purpose of acquiring the site and buildings of the Royal Infirmary and of making a capital contribution towards the provision of new infirmary accommodation by the board of management of the Bradford Royal Infirmary.

### A CHILDREN'S FRIEND.

By the death of Mr. George Gibson Johnson, at the advanced age of seventy-seven years, the Leicester Royal Infirmary loses one of its life governors who was particularly interested in children, and whose many acts of kindness will be remembered. For some few years Mr. Johnson was a member of the Board of Governors, but increasing deafness compelled his retirement. He was a supporter of the institution in many ways, and contributed from time to time to the special funds. But it is no less for his unostentatious gifts of eggs, fruit, and vegetables that he will be much missed. His concern for the children suggested to him that a weekly supply of fresh eggs would be invaluable, and week by week he motored in from the country with sometimes forty and sometimes fifty eggs, with the result that by the end of the year he had brought over 2,000 to the hospital. It is interesting to record that his will contains a bequest of £100 to the institution he so often helped.

### THE WAR AND MENTAL DISEASE.

One of the sad effects of the war, which as time goes on will require investigation, is seen in its influence on people liable to mental derangement. More than one mental hospital has already experienced strain, and in certain instances quite a rush of patients has been felt. Indeed, the war, working in different ways, has precipitated quite a number of unfortunates into various states of insanity, and medical officers, as we know from more than one mental institution, are recording quite a rush of work in consequence. It will be interesting to see if statistics are available as to the true extent of the war's influence in this matter, and also the particular nature of the derangements which it has occasioned. We shall be glad to open our columns to information on this head, which, though necessarily limited at present, may yet serve to indicate the direction of the tendency.

### TUBERCULOSIS HOSPITALS FOR THE WOUNDED.

The question of the provision of more adequate hospital accommodation (in view of the admitted need of the nation for in-patient treatment on a more extended scale) is one which the National Insurance Act had brought into prominence before the outbreak of war gave it a special emphasis through the arrival of wounded. The cases being largely light, at any rate so far, private offers and extemporised buildings have been sufficient to meet the need, but logically minded persons are asking whether there is not a better and more permanently useful solution. Thus a writer in *Kahncrete Engineering* makes the suggestion that the existing machinery of the National Insurance Act should be used to hurry forward the building of permanent consumption hospitals, which could be devoted to accommodating the wounded until the end of the war, after which they should revert to their original purpose. The writer contends that makeshift expedients would thereby be avoided, and the money spent would have ultimate as well as immediate practical value. He suggests, in short, that the Government should make a grant to those local authorities who will undertake to have their hospitals ready by a specified, and greatly hastened, date. The rest of the journal is devoted to a description of types of economical buildings and materials.

### THIS WEEK'S DRUG MARKET.

The improvement in the tone of the Drug market noticed last week has been maintained, and considering all the circumstances it is remarkable that business is proceeding so smoothly. Of course, nothing like normal conditions prevail, but there are undoubted signs that confidence in the market is being restored. For most of the synthetic drugs and fine chemicals very high prices continue to be quoted and must necessarily rule during the continuance of the war, notwithstanding the arrivals from neutral countries and the fact that some of them which have hitherto been obtained solely from the Continent are now being made here. It seems probable that still higher prices will be wanted for carbolic acid, as larger quantities must be required for purposes connected with the war. In consequence of an advance in the value of quicksilver, quotations for corrosive sublimate, calomel, and other salts of mercury have been raised substantially. Among the articles which have lately risen in price are the following: boric acid, borax, Epsom salts, and atropine. On the other hand, citric acid, tartaric acid, cream of tartar, balsam Peru, cocaine, essence of lemon, menthol, senega root, and bromides have a lower tendency in price. Opium has not recently changed to any appreciable extent, and the same applies to morphine, but codeine is dearer. Any increase in the demand for cod-liver oil would most probably be followed by an advance in price. The periodical public sales of drugs in Mincing Lane have been resumed after an interval of two and a half months; it is hardly expected that the demand at these auctions will be brisk, but it is satisfactory to know that confidence has been sufficiently restored to make it desirable to continue them.

### TO OUR READERS.

Contributions are specially invited from any of our readers to these columns. They should deal with topical subjects and news. They must be authenticated for the information of the Editor only. The minimum payment if published is 5s. There is no hard-and-fast rule as to space, but notes of about twenty lines in length are preferred.

